# Predicting Associations of miRNAs and Candidate Gastric Cancer Genes for Nanomedicine

**DOI:** 10.3390/nano11030691

**Published:** 2021-03-10

**Authors:** Aigul Akimniyazova, Anna Pyrkova, Vladimir Uversky, Anatoliy Ivashchenko

**Affiliations:** 1 Department of Biotechnology, Al-Farabi Kazakh National University, Almaty 050040, Kazakhstan; akimniyazova.aigul@med-kaznu.com (A.A.); anna.pyrkova@kaznu.kz (A.P.); 2 Department of Molecular Medicine, Morsani College of Medicine, University of South Florida, Tampa, FL 33612, USA; vuversky@usf.edu

**Keywords:** nanoscale miRNA, mRNA, genes, human, primates, gastric cancer, diagnostics, nanomedicine

## Abstract

Nanoscale miRNAs regulate the synthesis of most human proteins involved in differentiation, proliferation, cell cycle, apoptosis, and other processes associated with the growth and the development of an organism. miRNAs also play a number of important roles in the development of gastric cancer. In this work, we studied the quantitative characteristics of miRNA interactions with 69 candidate gastric cancer genes using bioinformatics approaches. To this end, the MirTarget program was used, which determines the characteristics of miRNA binding to mRNA in the 5′UTR, CDS, and 3′UTR. Associations of miRNAs with alternative target genes and associations of genes with alternative miRNAs were established. The cluster organization of miRNA binding sites (BSs) in mRNA was revealed, leading to the emergence of miRNA competition for binding to the mRNA of a target gene. Groups of target genes with clusters of overlapping BSs include miR-5095, miR-619-5p, miR-1273 family, miR-466, ID01030.3p-miR, ID00436.3p-miR, miR-574-5p, and ID00470.5p-miR. In the defined associations of target genes and miRNAs, miRNA BSs are organized into clusters of multiple BSs, which facilitate the design and the development of a system of chips that can be used to control the state of miRNA and target genes associations in gastric cancer.

## 1. Introduction

Endogenous gene expression regulators include nanoscale miRNAs (mRNAs inhibitory RNA), which are small single-stranded non-coding RNA molecules that directly or indirectly control the synthesis of most human proteins [1,2,3,4,5,6]. The miRNA target genes predominantly encode transcription factors and various kinases. Therefore, one can speak of the crucial role of miRNA in regulating genome expression as a whole. For more than 20 years, attempts have been made to use miRNA as diagnostic molecules and therapeutic agents for various diseases [7,8,9,10,11,12,13,14,15,16]. However, these attempts did not lead to the creation of the methods for diagnosing and treating the disease using miRNA, mostly due to the polygenic nature of most diseases, the inadequacy of the used bioinformatics approaches for determining the target genes of miRNA, the lack of possibility to unambiguously predict side effects of the proposed diagnostic and treatment methods, as well as a large amount of required materials, material costs, and others. Nevertheless, miRNAs continued to be studied for use in nanomedicine [17,18,19,20,21]. Furthermore, several publications, where the bioinformatics approach developed by us was applied, revealed that the aforementioned difficulties are surmountable [22,23,24,25,26,27,28,29].

The success in the development of nanomedicine depends on the progress in studying the molecular basis of diseases. Most diseases are caused by the impaired expression of many genes and many miRNAs [30,31,32,33]. Therefore, to get deeper insights into the molecular basis of diseases, it is necessary to study the regulation of all human genes by all human miRNAs of the expression. It is because the study of small samples of genes and miRNAs would not lead to a quick solution to the disease diagnosis and treatment issues due to the possible uncertainties and the lack of assessment of side effects. Using the examples set forth by earlier studies [22,23,27,28,29], in this work we propose the approaches for creating chips that help establish the stable associations of miRNAs and their target genes, which must be determined simultaneously for an unambiguous interpretation of the results.

Gastric cancer (stomach cancer) develops rapidly and, as a rule, even early diagnosis of the disease does not significantly affect the duration of the disease [30]. Added to this is the lack of effective treatments for gastric cancer. Although the information about miRNAs effect on the development of the disease is available in the long-standing literature, this knowledge has not led to the elucidation of which miRNAs can influence the expression of candidate genes for gastric cancer [31,32,33,34,35,36,37,38,39,40,41,42,43,44]. This is due to the inadequate methods for setting up the interaction of miRNA with candidate target genes. Typically, the literature describes correlations between changes in gene expression and miRNA. Such information is insufficient to suggest methods for the disease diagnosis using miRNA. In connection with these circumstances, there is an urgent need to determine which candidate gastric cancer genes can be affected by miRNAs, and how they can be used for the diagnosis and therapy of gastric cancer. For this to happen, it is necessary to control the quantitative characteristics of the interaction between miRNA and mRNA, since there is competition between several miRNAs when interacting with one or more target genes. In addition, some miRNAs can bind to mRNAs of several genes [24,26,27,28,29]. A systematic approach to considering the interaction of miRNA with target genes counts the following circumstances: (a) the target genes for one miRNA are alternative genes for this miRNA; (b) two or more miRNAs that have binding sites (BSs) in one gene are alternative miRNAs; (c) the BSs of alternative miRNAs can be located separately in the mRNA or organized into clusters with overlapping nucleotide sequences; (d) one miRNA can have multiple BSs with overlapping nucleotide sequences in the mRNA; and (e) two or more alternative miRNAs can also have multiple BSs.

Some genes cause different oncological diseases, and some miRNAs are involved in developing different types and subtypes of malignant neoplasms. Therefore, such diseases can be adequately diagnosed only using many miRNA associations and their target genes. This problem is complex and can be solved by considering the quantitative characteristics of the interaction between miRNA and mRNA. This can be helped using bioinformatics approaches, which greatly facilitate and reduce the cost of establishing associations between miRNA and target genes that adequately reflect the relationship between the expressions of these molecules. In this study, we analyze the quantitative characteristics of the interaction of miRNA and target genes, study the localization of BSs in the mRNA in the 5′UTR (5′-untranslated region), CDS (coding sequence), and 3′UTR (3′-untranslated region) of mRNA, and look for the associations of miRNA and gastric cancer candidate genes. The expressions of miRNA and target genes are considered in the normal human tissue types as well. The approach we are developing for identifying miRNA associations and their target genes creates the basis for the manufacture of the chips suitable for diagnosing the diseases caused by many genes and miRNAs.

## 2. Materials and Methods

The nucleotide (nt) sequences of candidate genes associated with gastric cancer were downloaded from the NCBI (http://www.ncbi.nlm.nih.gov. accessed on 10 May 2020). The nucleotide sequences of 2567 miRNAs (old miRNAs) were obtained from miRBase (http://www.mirbase.org/. accessed on 10 May 2020), and 3707 miRNAs (novel miRNAs) were taken from an earlier study [45]. The consensus normalized expression (NX) value for each gene was provided by the Human Protein Atlas data (https://www.proteinatlas.org. accessed on 20 May 2020). Orthologous genes of the following objects were used in the work: *Bos taurus* (bta), *Canis familiaris* (cfa), *Equus caballus* (eca), *Gorilla gorilla* (ggo), *Homo sapiens* (hsa), *Macaca fascicularis* (mfa), *Macaca mulatta* (mml), *Macaca nemestrina* (mne), *Mus musculus* (mmu), *Nomascus leucogenys* (nle), *Ovis aries* (oar), *Pongo abelii* (pab), *Papio anubis* (pan), *Pan paniscus* (ppa), *Pan troglodytes* (ptr), *Rattus norvegicus* (rno), *Rhinopithecus roxellana* (rro), *Saimiri boliviensis* (sbo), and *Sus scrofa* (ssc). The miRNA BSs in the 5′UTRs, CDSs, and 3′UTRs of several genes were predicted using the MirTarget program [46,47]. This program defines the following features of the miRNA binding to mRNAs: (a) the start of the initiation of miRNA binding to mRNAs; (b) the localization of miRNA BSs in the 5′UTRs, CDSs, and 3′UTRs of mRNAs; (c) the free energy of the interaction between miRNAs and mRNAs (ΔG, kJ/mole); and (d) the schemes of nucleotide interactions between miRNAs and mRNAs. The ratio ΔG/ΔGm (%) was determined for each site (ΔGm equals to the free energy of miRNA binding with its fully complementary nucleotide sequence). The miRNA BSs located in the mRNAs had ΔG/ΔGm ratios of 90% or higher. The ΔG/ΔGm ratios were determined on the assumption that the members of one miRNA family generally differed by no more than one to three nucleotides, and along with a miRNA length of 22 nt, the ΔG/ΔGm value was determined to be ranged from 91% (20 nt/22 nt = 91%) to 95% (21 nt/22 nt = 95%). With a larger difference in the number of mismatched nucleotides, the probability of two or more miRNAs binding to one site increases, despite the natural ability of miRNAs to interact selectively with the mRNAs of the target gene. The MirTarget program identifies the positions of the BSs on the mRNA, beginning with the first nucleotide of the mRNA’s 5′UTR. It also identifies hydrogen bonds between adenine (A) and uracil (U), guanine (G) and cytosine (C), G and U, as well as A and C. The distance between A and C was 1.04 nanometers; the distance between G and C and between A and U was 1.03 nanometers; and the distance between G and U was 1.02 nanometers [48]. The numbers of hydrogen bonds in the G-C, A-U, G-U, and A-C interactions were 3, 2, 1, and 1, respectively [48,49,50,51]. The MirTarget program determines single miRNA BSs in the mRNAs and miRNA BSs in clusters (i.e., arranged in series with overlapping nucleotide sequences of the same or several different miRNAs). In this study, we assumed that the miRNA BSs in mRNAs were organized into clusters.

## 3. Results

### 3.1. The Characteristics of the miRNA Interactions with 5′UTR mRNAs of Gastric Cancer Candidate Genes

The characteristics of the interaction of 6274 miRNA with mRNA of 69 candidate genes for gastric cancer were studied (Table S1). Table 1 shows the quantitative characteristics of miRNA binding in 5′UTR of mRNA genes having clusters of miRNA BSs. The *ARID1A* gene encoded protein is a part of the large ATP-dependent chromatin remodeling complex SNF/SWI, required for the transcriptional activation of genes normally repressed by chromatin formation. The mRNA of the *ARID1A* gene contains a cluster of ten miRNA BSs located from 136 nt to 192 nt. The sum of the lengths of the BSs of these miRNAs is 272 nt, which are located in a 57-nt cluster of BS. In other words, there is the 4.8-time compaction of BSs within this mRNA segment. It leads to the competition between miRNAs when they bind to a cluster. The advantage in binding will be shown by the miRNAs that interact with an mRNA with a higher free energy and present in higher concentrations compared to the competing miRNAs. The average free energy of interaction of all miRNAs in the 5′UTR mRNA of candidate genes (Table 1 and Table S2) is −126 ± 8 kJ/mole. On this basis, we propose a value of −130 kJ/mole and higher as a criterion for the effective interaction of miRNA with 5′UTR mRNA. To diagnose gastric cancer, we hypothesized that there are the associations of ID01778.3p-miR, ID00296.3p-miR, ID01702.3p-miR, and the ARID1A gene. Therefore, when developing a chip, it is necessary to use all nine miRNAs to consider the changes in their concentration, since this is an essential factor in the competitive interactions with mRNA. 

The nucleotide sequences of the cluster of the miRNA BSs at the *ARID1A* gene in *G. gorilla*, *H. sapiens*, *M. mulatta, M. nemestrina*, *N. leucogenys*, *P. anubis*, *P. paniscus,* and *P. troglodytes* are shown in Figure S1. Flanking oligonucleotides from the 5-end (GGGAGCAGCUG) and 3-end (GAGCCUGAGCCGG) are identical in all subjects. These data indicate the emergence and persistence of these miRNA and target gene associations for many millions of years. This makes it possible to elucidate the role of the candidate *ARID1A* gene in the studied subjects as models for gastric cancer.

The transcription factor E2F1 is involved in the development of a number of oncological diseases, including gastric cancer. The *E2F1* mRNA contains a 35 nt long cluster of the seven miRNAs BSs (Table 1), two of which bind with ΔG values above −130 kJ/mole, and the ID02052.5p-miR pair is completely complementary with high free energy. The sum of the BSs of seven miRNAs is 148 nt and is 4.2 times the cluster length. The associations of these miRNAs and the *E2F1* gene can serve as markers for the gastric cancer diagnosis. One of the ways to confirm the reality of the binding between miRNA and target genes is to check for the presence of these BSs in the orthologous genes. Figure S2 shows the nucleotide sequences of the cluster of BSs in mRNA of the orthologous *E2F1* genes. Flanking oligonucleotides from the 5-end (GGCCUGCCGC) and 3-end (GGCCGCGCGG) are identical in all objects. Substitutions of C for U practically do not affect the free energy of interaction between miRNA and mRNA, since non-canonical pairs G-U are formed instead of G-C.

The mRNA of the *ODC1* gene has the cluster of 15 miRNA BSs from 9 nt to 46 nt (Table 1). This cluster (38 nt) length is 10.6 times shorter than the sum of all BSs. Therefore, only one miRNA out of 15 miRNAs can bind to an mRNA. Such miRNA can be one of ID01804.3p-miR, ID02084.3p-miR, ID02064.5p-miR, and ID01702.3p-miR, which can interact with a ΔG value above −130 kJ/mole. If other miRNAs are present at concentrations significantly higher than the concentrations of these four miRNAs, they will have a final effect on the translation of mRNA of the *ODC1* gene.

The mRNA of the *PIK3CA* gene has a cluster of 12 miRNA BSs of 33 nt in length, which is 8.5 times less than the sum of the lengths of the BSs of these miRNAs, which is equal to 282 nt. Such compaction of the BSs leads to high competition between these miRNAs. Six out of 12 miRNAs have the free energy of interaction above −130 kJ/mole. This gives grounds to recommend considering the association of these six miRNAs with the *PIK3CA* gene as markers for gastric cancer diagnosis.

The mRNA of the *TBC1D9* gene also has a cluster of seven miRNAs, 37 nt long, which is five times shorter than the sum of the lengths of the BSs of these miRNAs, and therefore only one miRNA can bind to it. In the clusters of miRNA BSs of *TBC1D9* orthologous genes, a CGC insert in humans was detected (Figure S3). The 3-end flanking oligonucleotides (AGCGGACGGG) are identical across all objects. The flanking oligonucleotides of the 5-end are different, which indicates the functional features of the cluster of BSs and flanking nucleotide sequences.

For the objective assessment of the result of interaction between miRNA and mRNA, it is necessary to know both the quantitative characteristics of their interaction and the ratio of these molecules’ concentrations. This knowledge is necessary for predicting their interactions based on the analysis of the effect of the inhibitor (miRNA) on the activity of the translation process. One miRNA, which has one BS, can bind to mRNA reversibly when their interaction is short-lived, or irreversibly when the bond is relatively long-term, or can promote mRNA degradation. The result of the interaction of miRNA with mRNA will depend on the ratio of miRNA and mRNA concentrations. An increase in miRNA concentration will proportionally decrease protein synthesis, that is, at a ratio of miRNA to mRNA concentration of 1:10, protein synthesis will decrease only by 10%, and at a ratio of 10:1, protein synthesis will be suppressed by 90%. Therefore, it is necessary to determine the ratio of the concentrations of miRNA and mRNA gene using chips.

### 3.2. The Characteristics of the miRNA Interactions with CDS mRNAs of Gastric Cancer Candidate Genes

The mRNA of the *ARID1A* gene contains three clusters of BSs in the protein coding region (Table 2). The first cluster is located from 410 nt to 442 nt and includes the BSs of eight miRNAs. The cluster length is 32 nt, which is 5.8 times less than the sum of the lengths of all BSs (184 nt). This compaction leads to the fact that only one miRNA can bind in a cluster. Three out of eight miRNAs bind with a ΔG value equal to or greater than −130 kJ/mole, which indicates a strong interaction of the members of associations of these miRNAs with the ARID1A gene.

The nucleotide sequences of the cluster of BSs are identical in the mRNA of the orthologous genes *G. gorilla*, *H. sapiens*, *M. mulatta*, *M. nemestrina*, *N. leucogenys*, *P. anubis*, *P. paniscus*, and *P. troglodytes* (Figure S4). Flanking oligonucleotides are also identical at the 5-end and 3-end of all objects. The oligopeptide LGNPPPPPPS encoded by the clusters of BSs is also identical in all ARID1A orthologous proteins. The location of the miRNA BSs in the 5′UTR and at the beginning of the CDS leads to an early arrest of protein synthesis, which saves energy compared to the termination of protein synthesis at the end and a higher probability of the formation of an abortive protein.

The second cluster in the mRNA of the ARID1A gene consists of ID01704.5p-miR and ID02761.3p-miRBSs, which has two BSs with a ΔG value of more than −130 kJ/mole. The third cluster includes BSs of three miRNAs with a free interaction energy miRNA of −125 kJ/mole and more. Therefore, the synthesis of the ARID1A protein is highly dependent on the expression of 13 miRNA binding in 5′UTR mRNA. Since this protein performs an vital function in cells, the miRNA concentration must be significantly lower than the mRNA concentration for the protein to be synthesized in the required amount.

The protein E2F1 is a member of the E2F family of transcription factors and plays a crucial role in the control of cell cycle and action of tumor suppressor proteins. The mRNA of the *E2F1* gene contains a cluster of BSs for three miRNAs, of which ID02051.3p-miR binds complementarily to all nucleotides with a high ΔG value (Table 2). Therefore, the association ID02051.3p-miR binding in CDS mRNA of the gene is recommended for gastric cancer diagnosis. Figure S5 shows the nucleotide sequences of the mRNA regions of orthologous *E2F1* genes containing clusters of BSs of three miRNAs. Flanking nucleotide sequences at the 5-end and 3-end are conserved. In *H. sapiens*, *G. gorilla*, and *P. anubis*, the clusters of BSs encode the PAAPAAGP oligopeptide; in *P. troglodytes* and *P. paniscus*, this oligopeptide is also encoded despite the substitution of C for U; and in *P. paniscus*, *M. mulatta*, *M. fascicularis*, and *M. nemestrina* encoding also occurs when replacing C with A in the third position of the CCC codon. In N. leucogenys, the cluster of BSs encodes the PTAPAAGP oligopeptide, since G is replaced by A at the first codon position. In all cases of nucleotide substitutions, canonical pairs are replaced by non-canonical pairs and, therefore, single nucleotide polymorphisms practically do not change the affinity of miRNA to mRNA of the target gene.

The MAPK1 protein is involved in proliferation, differentiation, transcription regulation, and development. CDS mRNA of the *MAPK1* gene contains a compact cluster 28 nt long, which is 2.9 times less than the sum of the BSs of all miRNAs (Table 2). Of the four miRNAs, three bind with a ΔG value of more than −130 kJ/mole. These characteristics of the associations of three miRNAs with the *MAPK1* gene make it possible to recommend them for gastric cancer diagnosis. The cluster of BSs in the mRNA of the human MAPK1 gene (Figure S6) encodes an oligopeptide AAAAAAGAG, identical in *P. anubis*, *P. paniscus*, *P. troglodytes*, *M. mulatta*, *M. nemestrina*, *N. leucogenys*, *P. abelii*, and *G. gorilla*. In *P. abelii* and *G. gorilla*, the clusters encode the longer oligopeptides AAAAAAAAAGAG and AAAAAAAAAAGAG, respectively. In mRNA of the mouse and rat *MAPK1* gene, the 22 nt clusters contained a sequence of GCG triplets that could bind ID03332.3p-miR and encode AAAAAAG. The results presented clearly demonstrate the variability of the cluster length of BSs compared to the flanking nucleotide sequences, which are highly conserved.

The *TERT* gene encodes telomerase that plays a role in cellular aging. The deregulation of telomerase expression in somatic cells may be involved in oncogenesis. The CDS mRNA of the *TERT* gene contains a cluster of BSs of three miRNAs binding with high free energy (Table 2). The nucleotide sequence of the cluster of BSs is conserved in the CDS mRNA of the *TERT* gene (Figure S7) and encodes the LGAKGAAGPLPS oligopeptide in all TERT orthologous proteins of the studied objects.

The CDS mRNA of the human *VEGFC* gene has a cluster of seven miRNA BSs (Table 2). The cluster of these miRNA BSs has a length of 31 nt, which is 5.1 times less than the sum of BSs. The *VEGFC* gene BSs is the longest in *M. nemestrina* and encodes the REAPAAAAAAALE oligopeptide. In other objects, the cluster length decreases due to the GCC codon, but remains sufficient for binding ID02052.5p-miR, ID02187.5p-miR, ID01041.5p-miR, and ID01873.3p-miR. For the flanking nucleotides, the cluster of BSs are conserved in the mRNA of the *VEGFC* gene of all objects (Figure S8).

### 3.3. The Characteristics of miRNA Interactions with 3′UTR mRNAs of Gastric Cancer Candidate Genes

**Figure 1:**
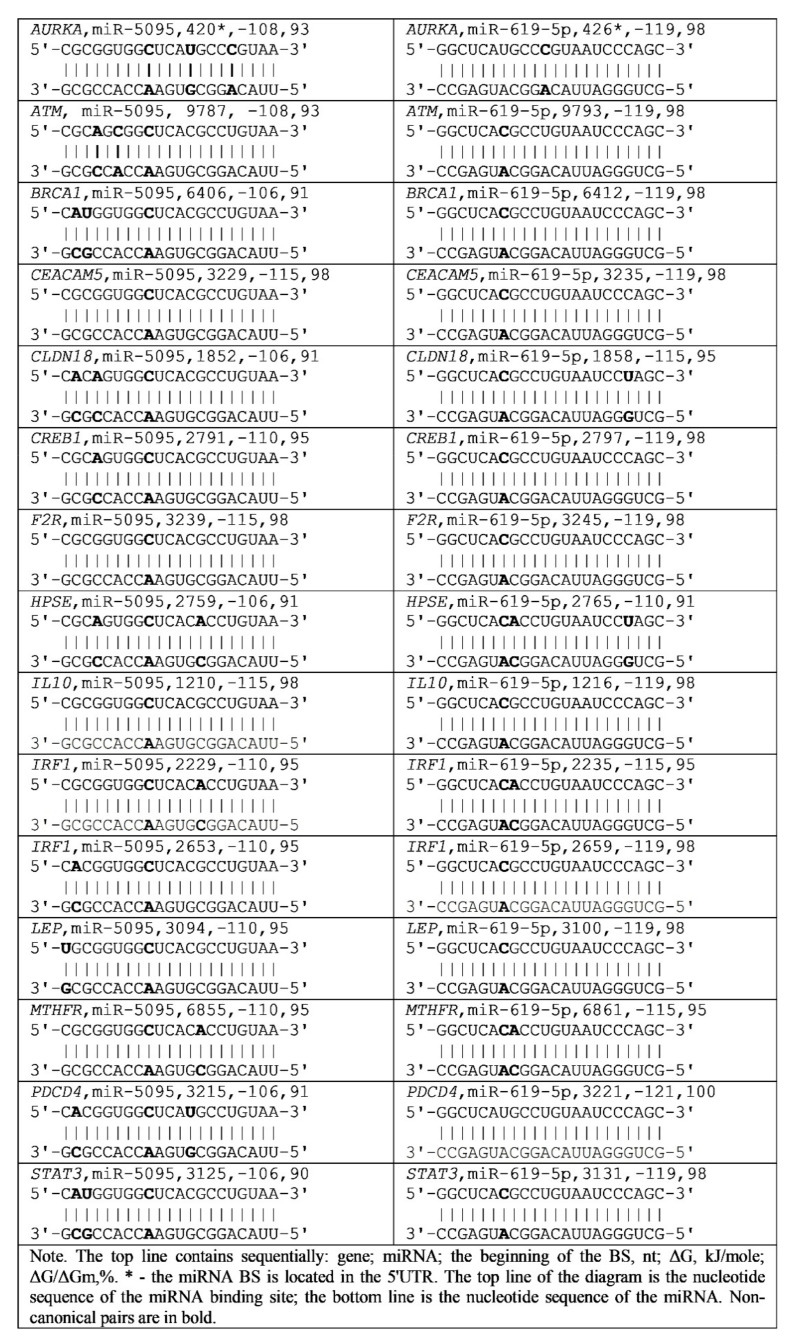
Schemes of interaction of miR-5095 and miR-619-5p in the 3′UTR of mRNA of candidate gastric cancer genes.

The 3′UTR of mRNAs of gastric cancer candidate genes contains multiple BSs for several miRNAs (Table 3 and Table S4). A feature of 14 candidate gastric cancer genes is the presence of miR-5095 and miR-619-5p BSs in their 3′UTR mRNA with overlapping nucleotide sequences (Figure 1). The schemes of the interaction of miR-5095 and miR-619-5p with the mRNA of these genes indicate a high complementarity of nucleotides, including non-canonical A-C and G-U bonds.

The presented schemes for the interaction of miR-5095 and miR-619-5p in the 3′UTR of mRNA genes indicate the high efficiency of the MirTarget program in determining the quantitative characteristics of the interaction of miRNA with mRNA. Both miRNAs in all cases, considering non-canonical nucleotide pairs along the entire length, complementarily bind to mRNA (Figure 1). All 14 genes in the combination with miR-5095 and miR-619-5p are recommended as the associations for gastric cancer diagnosis.

The nucleotide sequences of the cluster of miR-5095 and miR-619-5p BSs are mostly conserved (Table S4), except for the positions in which there are substitutions of purine for purine (G 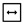 A) and pyrimidine for pyrimidine (C 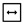 U), which have little effect on the free energy of miRNA interaction with mRNA. The data presented indicate the stability of the interactions of these miRNAs with the mRNA of candidate genes for gastric cancer.

**Figure 2:**
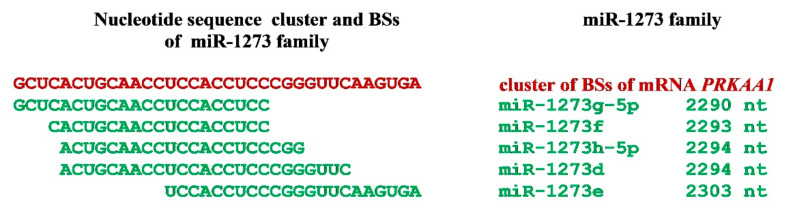
The scheme of miR-1273 family binding sites (BSs) in the 3′UTR of the mRNA of the candidate *PRKAA1* gene.

The *PRKAA1* gene is a target of miR-1273d, miR-1273e, miR-1273g-5p, miR-1273f, and miR-1273h-5p (Figure 2 and Table S4). The BSs of these miRNAs, except miR-1273g-3p, form a cluster, conserved in the mRNA of the orthologous *PRKAA1* genes in *P*. *troglodytes*, *G. gorilla*, *P. abelii*, *P. anubis*, *P. paniscus*, *N. leucogenys*, *M. mulatta*, and *M. nemestrina*. The miR-1273 family with the *PRKAA1* gene forms the association recommended for gastric cancer diagnosis.

The miRNAs of the miR-1273 family had BSs in the mRNA of 12 gastric cancer candidate genes (Table 3 and Table S4). Considering the formation of non-canonical nucleotide pairs, almost all nucleotides of each miRNA formed hydrogen bonds with the nucleotides of the mRNA (Figure S9). Some miRNA families had BSs in mRNA with overlapping nucleotide sequences, that is, they had clusters of BSs. For example, miR-1273a and miR-1273c competitively bind to the mRNA of the *ATM*, *CDH1*, *POU5F1*, *SGCB*, and *TP53* genes. The miR-1273e and miR-1273f have overlapping mRNA BSs for the *ATM*, *KRAS*, *MACC1*, *MDM2*, *POU5F1*, *PRKAA1*, and *SGCB* genes. The mRNAs of *CDH17*, *MACC1*, *POU5F1*, and *PRKAA1* genes contain a cluster of BSs for miR-1273d and miR-1273f. The above associations of miRNAs of the miR-1273 family and candidate target genes for gastric cancer are recommended for the diagnosis of this disease.

In the 3′UTR mRNA of five genes, there are the clusters of BSs for miR-466, ID01030.3p-miR, and ID00436.3p-miR consisting of two or more repeats of GU dinucleotides (Table 3 and Table S4). The interaction schemes of these miRNAs with the mRNAs of target genes show almost complete complementarity with the formation of canonical and non-canonical nucleotide pairs (Figure S10). The associations of miR-466, ID01030.3p-miR, and ID00436.3p-miR with five candidate target genes for gastric cancer are proposed to be used as diagnostic markers of this disease.

Among the candidate genes for gastric cancer, 6 target genes were identified for miR-574-5p and ID00470.5p-miR (Table 3 and Table S4). The interaction schemes of these miRNAs with mRNA of target genes are shown in Figure S11. The cluster of BSs of these miRNAs consist of repeats of the CA dinucleotide in the mRNA of the *IGF2* gene from 2 to 10 times in the mRNA of the gene. The miR-574-5p and ID00470.5p-miR associations, together with target genes, are recommended to use for gastric cancer diagnosis

In addition to the associations of miRNA and mRNA genes contained in the clusters, there are associations of the miRNA and genes not included in the clusters. From Tables S1 and S3, such associations for which the free energy of interactions miRNA and mRNA is −130 kJ/mole and above are as follows: ID02052.5p-miR and *DNMT1*; ID02457.3p-miR and *EGFR*; ID02761.3p-miR and *EZH2*; ID03047.3p-miR and *FGFR2*; miR-6789-5p and *HIF1A*; ID03332.3p-miR and *KRAS*; ID02064.5p-miR and *NFKB1*; ID02761.3p-miR and *PTEN*; ID02064.5p-miR and *TGFB1*; ID00252.5p-miR, ID00961.3p-miR, ID00049.5p-miR and *TIMP2*; ID02294.5p-miR and *ACE*; ID01895.5p-miR, ID02052.5p-miR and *CDX2*; ID00920.5p-miR and *OGG1*; miR-4767, ID03332.3p-miR and *SIRT1*. 

In the 3′UTR mRNA of candidate gastric cancer genes, the miRNA and genes associations are characterized by the free energy of interaction of −115 kJ/mole and above (Table S4). These interactions include miR-619-5p and *BRCA1*; miR-1273d, miR-1273h-5p (100%) and *CASP10*; miR-1273a, miR-1273d and *CDH17*; miR-5095, miR-619-5p and *CEACAM5;* miR-619-5p and *ERBB3*; ID03224.5p-miR and *KRAS*; miR-619-5p and *MACC1*; ID01404.5p-miR, ID02732.3p-miR and *SMAD4*; miR-6089, ID03306.3p-miR, ID03208.5p-miR, ID00296.3p-miR, miR-1183 and *TGFB1*; ID01941.5p-miR and *TIMP2*; ID00548.3p-miR, ID02379.3p-miR and *TP53.*

Table 4 provides information on the miRNAs targeting two or more candidate genes. ID02064.5p-miR has six alternative target genes with free energies of interaction ranging from −129 to −140 kJ/mole. miR-3960 and ID02052.5p-miR each have four alternative target genes. The *ARID1A* gene interacts with six alternative miRNAs. The expression of the *VEGFC* gene can depend on four alternative miRNAs, while the expression of the *TBC1D9* gene can depend on three miRNAs. The information given in Table 4 shows that the aforementioned associations of miRNA and candidate genes require the development of a validation system of the obtained results, which will allow for identifying the associations that reflect most efficiently the state of the disease. Considering the identified associations of miRNA and target genes organized into clusters and multiple binding sites, it becomes clear that it is vital to create a system of chips that will control and reflect the state of the associations between the miRNAs and target genes in disease.

## 4. Discussion

The current work has found the associations of one miRNA and one gene; several miRNA BSs which form a cluster in the mRNA of one gene; several miRNAs and several genes, and others. Such a variety of the associations of miRNAs and target genes is a consequence of the polygenic nature of a number of diseases, including gastric cancer. Numerous attempts to identify individual miRNAs as markers of such diseases have been unsuccessful for numerous reasons. Approximately half of the miRNAs are of intronic origin (as a rule, this is not even mentioned) and are co-expressed with the host gene, that is, they can be a consequence of a change in the expression of a gene involved in the disease development but not in the cause of the disease. Therefore, it is necessary to signify the expression of both the miRNAs of target genes and the host gene. Based on the stated reasons, the established correlations between changes in miRNAs concentration and signs of the disease are hypothetical and require verification since the development of therapy methods on such a basis is almost impossible. The establishment of many associations, each of which can serve as an indicator of the disease, significantly complicates the selection of high selectivity markers that determine the disease and the degree of its development. Therefore, it is necessary to highlight the associations most responsible for the disease, which is not easy due to the various forms and characteristics of the disease. It is essential simultaneously monitor the changes in the members of the associations (miRNAs or mRNAs) in order to identify the leading components since the member of the association can be the member of one or more associations. This becomes clear from the data in Table 4. For example, ID02064.5p-miR can bind to mRNA of six genes, and six miRNAs can target the *ARID1A* gene. Even this simple example demonstrates the need to analyze many associations of miRNAs and target genes to establish their involvement in the disease. The simultaneous determination of the concentration of miRNA and mRNA using chips will make it possible to establish which of the miRNA and mRNA association members is responsible for the disease development and to what extent. For example, an increase in miRNA concentration will lead to a decrease in mRNA translation. If protein synthesis changes, with a constant miRNA concentration, it is necessary to correct the expression of the gene by encoding the protein. A change in miRNA concentration can lead to a decrease (increase) in protein synthesis, and therefore miRNA is the primary cause of the disease.

The comparison of the NX values of target genes with the NX of host genes (co-expressed with miRNA) shows that they are comparable, which allows for maintaining the relationship of the association components in approximately equal proportions. There is a tendency that highly expressed genes do not have clusters of miRNA binding sites in the mRNA (Table 1, Table 2 and Table 3 and Tables S2–S4). Only one to three binding sites were identified in their mRNAs, usually located separately from each other. For example, mRNA (the NX value is indicated in parentheses) of *CDKN1A* (25.2), *SMAD2* (26.3), *LGALS3* (36.9), *JUN* (47.0), *MUC6* (76.6), and *PGC* (963.7) genes each have one binding site. The mRNA of the *ERBB3* (32.9) and *MUC1* (105.0) genes each have two binding sites, and the mRNA of the *PDCD4* (27.6), *STAT3* (29.4) *THBS1* (46.5), *MUC6* (76.6), and *CLDN18* (126.9) genes have three miRNA binding sites.

The knowledge of the expression of host genes of intronic miRNAs helps predict the preferential binding of competing miRNAs not only by the free interaction energy but also by their concentration. For example, the free binding energy of miR-619-5p equal to −121 kJ/mole is greater than ΔG equal to −117 kJ/mole for miR-5095, but miR-5095 is expressed 4.8 times more than miR-619-5p, which gives it an advantage in binding in the miR-619-5p and miR-5095 BSs clusters. It is possible that only significant changes in the concentration of miRNA or mRNA of genes of associations can significantly change their interactions, which will lead to the emergence of pathology.

## Figures and Tables

**Table 1:** The characteristics of the miRNA interactions with 5′UTR mRNAs of gastric cancer candidate genes.

Gene; NX	miRNA	Start of Site, nt	ΔG, kJ/mole	ΔG/ΔGm, %	Length, nt
*ARID1A*; 19.5	ID02106.3p-miR	136	−123	89	23
	ID01778.3p-miR	140	−134	90	24
	ID00296.3p-miR	141, 166	−138	88	25
	miR-6081	143	−125	89	24
	ID01702.3p-miR	147	−134	89	24
	ID00465.5p-miR	148	−113	93	20
	ID01377.3p-miR	152	−117	92	20
	ID02592.5p-miR	164, 167	−123	89	23
	miR-3960	167	−115	92	20
	ID03065.3p-miR	172	−115	92	21
*E2F1*; 3.4	ID02574.3p-miR	84	−115	93	20
	ID02052.5p-miR	85	−149	100	24
	ID01873.3p-miR	87	−123	94	21
	miR-3960	90	−115	92	20
	ID00071.3p-miR	90	−117	92	20
	ID00722.5p-miR	90	−113	93	20
	ID02064.5p-miR	95	−132	91	23
*ODC1*; 19.2	ID00756.3p-miR	9	−129	94	23
	ID01804.3p-miR	13	−132	90	23
	ID02187.5p-miR	14	−123	89	23
	ID00457.3p-miR	15, 21	−123, −125	91, 92	22
	ID02084.3p-miR	17	−140	93	24
	ID02064.5p-miR	17 ÷ 23(3)	−129 ÷ −140	90 ÷ 97	23
	miR-3960	18 ÷ 21(3)	−115	92	20
	ID01652.3p-miR	19	−125	89	23
	ID02538.3p-miR	19	−123	92	22
	ID01702.3p-miR	19, 22	−134, −142	89, 94	24
	ID02229.3p-miR	21	−121	92	21
	ID02499.3p-miR	21	−119	92	21
	ID01157.5p-miR	22	−117	93	20
	ID01377.3p-miR	23	−121	95	20
	ID00061.3p-miR	24	−125	91	22
*PIK3CA*; 10.6	ID00296.3p-miR	1	−134	85	25
	ID01190.5p-miR	4	−140	92	24
	ID01702.3p-miR	4	−140	93	24
	ID01895.5p-miR	4	−132	89	24
	ID01641.3p-miR	5	−127	86	24
	ID00966.5p-miR	6	−136	90	24
	ID00030.3p-miR	7	−121	90	22
	ID02294.5p-miR	7	−125	86	24
	ID01804.3p-miR	8	−134	91	23
	ID01873.3p-miR	8	−123	94	21
	ID02064.5p-miR	9	−127	88	23
	ID02084.3p-miR	9	−129	86	24
*TBC1D9*; 10.0	ID01895.5p-miR	125	−134	90	24
	ID02187.5p-miR	130	−127	92	23
	ID03229.5p-miR	130, 133	−121	90	22
	ID01041.5p-miR	132	−132	90	24
	ID01702.3p-miR	136	−140	93	24
	ID02084.3p-miR	137	−136	90	24
	ID00457.3p-miR	138	−125	92	22

**Table 2:** The characteristics of miRNA interactions with CDS mRNAs of gastric cancer candidate genes.

Gene; NX	miRNA	Start of Site, nt	ΔG, kJ/mole	ΔG/ΔGm, %	Length, nt
*ARID1A*; 19.5	ID02052.5p-miR	410	−132	89	24
	ID00522.5p-miR	410	−127	91	23
	ID02187.5p-miR	411	−127	92	23
	ID02692.5p-miR	413	−127	90	23
	ID00457.3p-miR	415	−125	92	22
	ID02064.5p-miR	417	−134	93	23
	ID02084.3p-miR	418	−138	92	24
	ID02538.3p-miR	420	−125	94	22
	ID01704.5p-miR	471	−123	89	23
	ID02761.3p-miR	487, 493	−134, −140	90, 94	24
	ID00756.3p-miR	843	−125	91	23
	ID02294.5p-miR	851	−129	88	24
	ID00061.3p-miR	852	−125	91	22
*E2F1*; 3.4	ID02051.3p-miR	291	−153	100	24
	ID03448.3p-miR	292	−123	91	22
	ID01157.5p-miR	295	−117	93	20
*MAPK1*; 17.2	ID03332.3p-miR	243	−134	90	24
	ID01310.3p-miR	244	−121	92	22
	ID00798.3p-miR	246	−136	91	24
	ID01546.5p-miR	246	−132	90	24
*TERT*; 0.4	ID01098.3p-miR	3224	−125	89	24
	ID01338.5p-miR	3236	−132	91	24
	ID01816.3p-miR	3237	−138	92	24
*VEGFC*; 0.4	ID02052.5p-miR	498	−132	89	24
	ID02187.5p-miR	500	−123	89	23
	ID01041.5p-miR	501	−132	90	24
	ID01873.3p-miR	501	−123	94	21
	ID00457.3p-miR	503	−127	94	22
	miR-3960	504	−115	92	20
	ID02064.5p-miR	505	−129	90	23

**Table 3:** Characteristics of miRNA interactions with 3′UTR mRNAs of gastric cancer candidate genes.

Gene; NX	miRNA	Start of Site, nt	ΔG, kJ/mole	ΔG/ΔGm, %	Length, nt
*ATM*; 8.9	ID03006.5p-miR	9778	−121	89	24
	miR-5095	9787	−108	93	21
	miR-619-5p	9793	−119	98	22
	miR-5096	9882	−104	92	21
	miR-5585-3p	9950	−110	95	22
	miR-1273a	11,054	−119	90	25
	miR-1273g-3p	11,076	−113	96	21
	miR-1273e	11,119	−108	93	22
	miR-5585-5p	11,156	−106	91	22
*FLT1*; 4.6	ID01030.3p-miR	6909 ÷ 6923 (7)	−108 ÷ −110	89 ÷ 91	23
	miR-466	6911 ÷ 6937 (9)	−106 ÷ −108	89 ÷ 93	23
	ID00436.3p-miR	6913 ÷ 6925 (7)	−104	89	23
*IGF1*; 5.9	ID00470.5p-miR	4042 ÷ 4058 (9)	−108	89	23
	miR-574-5p	4042 ÷ 4062 (11)	−108 ÷ −113	89 ÷ 93	23
	miR-1273g-3p	6009	−113	96	21
	miR-1273f	6042	−102	98	19
	miR-1273d	6043	−119	87	25
	miR-1273e	6052	−108	93	22
*IGF2*; 3.2	ID00470.5p-miR	2286 ÷ 2351 (6)	−108 ÷ −113	89 ÷ 93	23
	miR-574-5p	2288, 2290	−108	89	23
	miR-574-5p	2397 ÷ 2408 (4)	−108 ÷ −113	89 ÷ 93	23
	ID00470.5p-miR	2404, 2412	−110	91	23
	ID00470.5p-miR	2442 ÷ 2463 (3)	−108 ÷ −113	89 ÷ 93	23
	miR-574-5p	2465, 2484	−108	89	23
	ID00470.5p-miR	2520 ÷ 2539 (3)	−108	89	23
	miR-574-5p	2522	−108	89	23
	ID00470.5p-miR	2655 ÷ 2672 (3)	−108 ÷ −110	89 ÷ 91	23
	ID00470.5p-miR	2704, 2725	−108	89	23
	miR-574-5p	2727, 2731	−108, −110	89, 91	23
*JAK2*; 14.1	miR-466	5182 ÷ 5200 (10)	−104 ÷ −106	89 ÷ 91	23
	ID01030.3p-miR	5184 ÷ 5200 (9)	−108	89	23
	ID00436.3p-miR	5184 ÷ 5202 (10)	−104 ÷ −106	89 ÷ 91	23
*SP1*; 18.8	miR-466	4145 ÷ 4161 (9)	−104 ÷ −106	89 ÷ 91	23
	ID01030.3p-miR	4147 ÷ 4159 (7)	−108	89	23
	ID00436.3p-miR	4147 ÷ 4161 (8)	−104 ÷ −106	89 ÷ 91	23
*XRCC1*; 17.2	miR-574-5p	2033 ÷ 2055 (11)	−108 ÷ −113	89 ÷ 93	23
	ID00470.5p-miR	2035 ÷ 2055 (9)	−108 ÷ −113	89 ÷ 93	23
*ZEB1*; 14.8	miR-574-5p	3587 ÷ 3605 (10)	−113	93	23
	ID00470.5p-miR	3587 ÷ 3605 (10)	−108	89	23

**Table 4:** The association of miRNA and alternative candidate genes for gastric cancer.

miRNA	Candidate Genes
ID02064.5p-miR	*ARID1A*, *E2F1*, *NFKB1*, *ODC1*, *TDFB1*, *VEGFC*
miR-3960	*ARID1A*, *CDX2*, *E2F1*, *ODC1*, *VEGFC*
ID02052.5p-miR	*CDX2*, *DNMT1*, *E2F1*, *VEGFC*
ID01041.5p-miR	*CDX2*, *TBC1D9*, *VEGFC*
ID02761.3p-miR	*ARID1A*, *EZH2*, *PTEN*
ID01702.3p-miR	*ARID1A*, *PIC3CA*, *TBC1D9*
ID01895.5p-miR	*PIC3CA*, *CDX2*, *TBC1D9*
ID03332.3p-miR	*KRAS*, *MAPK1*, *SIRT1*
ID00296.3p-miR	*ARID1A*, *TGFB1*
ID02084.5p-miR	*ARID1A*, *ODC1*

## Data Availability

Data is contained within the present article.

## Supplementary Materials

The following are available online at https://www.mdpi.com/2079-4991/11/3/691/s1, Figure S1: The nucleotide sequences of mRNA regions of orthologous *ARID1A* genes containing clusters of BSs of ten miRNAs, Figure S2: The nucleotide sequences of mRNA regions of orthologous *E2F1* genes containing clusters of BSs of three miRNAs, Figure S3: The nucleotide sequences of mRNA regions of orthologous *TBC1D9* genes containing clusters of seven miRNA binding sites, Figure S4: The nucleotide sequences of mRNA regions of orthologous *ARID1A* genes containing clusters of eight miRNA binding sites, Figure S5: The nucleotide sequences of CDS mRNA regions of orthologous *E2F1* genes containing clusters of BSs of three miRNAs, Figure S6: The nucleotide sequences of mRNA regions of orthologous *MAPK1* genes containing clusters of BSs of four miRNAs, Figure S7: The nucleotide sequences of mRNA regions of orthologous *TERT* genes containing clusters of BSs of three miRNAs, Figure S8: The nucleotide sequences of CDS mRNA regions of orthologous *VEGFC* genes containing clusters of seven miRNA binding sites, Figure S9: The schemes of miR-1273 family interaction in the 3’UTR of mRNA of candidate gastric cancer genes, Figure S10: The schemes of miR-466, ID01030.3p-miR, ID00436.3p-miR interaction with 3’UTR mRNA of gastric cancer candidate genes, Figure S11: The schemes of interaction of miR-574-5p and ID00470.5p-miR in the 3’UTR of mRNA of candidate gastric cancer genes. Table S1: The list of candidate gastric cancer genes, Table S2: The characteristics of the miRNA interactions with 5′UTR mRNAs of gastric cancer candidate genes, Table S3: The characteristics of the miRNA interactions with CDS mRNAs of gastric cancer candidate genes, Table S4: The characteristics of the miRNA interactions with 3’UTR mRNAs of gastric cancer candidate genes.
