# Supplementary material for: Predicting Associations of miRNAs and Candidate Gastric Cancer Genes for Nanomedicine
Source: Nanomaterials (Basel). 2021 Mar 10;11(3):691. doi: 10.3390/nano11030691 (PMC8000878; doi:10.3390/nano11030691)
Supplement: Supplementary file 1 [file nanomaterials-11-00691-s001.pdf]

Supplementary Materials to

Predicting Associations of miRNAs and Candidate Gastric Cancer Genes for Nanomedicine

Aigul Akimniyazova [1], Anna Pyrkova [1], Vladimir Uversky [2] and Anatoliy Ivashchenko [1,*]

[1] Department of Biotechnology, Al-Farabi Kazakh National University, Almaty 050040, Kazakhstan; akimniyazova.aigul@med-kaznu.com (A.A.); anna.pyrkova@kaznu.kz (A.P.)
[2] Department of Molecular Medicine, Morsani College of Medicine, University of South Florida, Tampa, FL 33612, USA; vuversky@usf.edu
* Correspondence: a.iavashchenko@gmail.com

Table S1. The list of candidate gastric cancer genes.

Gene	PMID	Gene	PMID	Gene	PMID
ACE	18059164	*HIF1A*	26339797	*PDCD4*	27021515
AQP3	26768614	*HPSE*	31257534	*PGC*	25549793
ARID1A	24744582	*IGF1*	29731870	*PIK3CA*	26543351
ATM	24841718	*IGF2*	24451943	*POU5F1*	30628031
AURKA	31871591	*IGFBP3*	24451943	*PRKAA1*	30253744
BRCA1	25266802	*IL10*	26088449	*PTEN*	26753960
CASP10	31611962	*IRF1*	27866197	*SFRP1*	23943784
CCND1	31982864	*JAK2*	25055044	*SIRT1*	24107295
CDH1	19168852	*JUN*	22002117	*SMAD2*	31773690
CDH17	24465527	*KRAS*	31217166	*SMAD4*	28440445
CDKN1A	28571676	*LEP*	33061532	*SP1*	24613927
CDX2	29113265	*LGALS3*	24971481	*STAT3*	32545648
CEACAM5	26374829	*MACC1*	26919111	*TBC1D9*	29137307
CLDN18	24073219	*MAPK1*	29550487	*TERT*	28423629
CREB1	25825983	*MDM2*	32023766	*TGFB1*	25118995
DNMT1	25595591	*MMP2*	24072772	*THBS1*	26075074
E2F1	32376557	*MTHFR*	25998065	*TIMP2*	24440352
EGFR	27997901	*MUC1*	24810688	*TIMP3*	27383203
EPHB2	32226496	*MUC6*	24816253	*TP53*	27323394
ERBB3	30621788	*NFKB1*	26801246	*TYMS*	28056823
EZH2	30952377	*ODC1*	28096401	*VEGFC*	29393383
FGFR2	26516773	*OGG1*	21822670	*XRCC1*	26768611
FLT1	24398900	*PARP1*	26540566	*ZEB1*	22466758

Table S2: The characteristics of the miRNA interactions with 5′UTR mRNAs of gastric cancer candidate genes

Gene; NX	miRNA	Start of site, nt	ΔG, kJ/mole	ΔG/ΔGm, %	Length, nt
AURKA; 4.3	miR-5095	420	-108	93	21
	miR-619-5p	426	-119	98	22
DNMT1; 9.0	ID02052.5p-miR	137	-134	90	24
EGFR; 7.8	ID02457.3p-miR	89	-132	95	22
EZH2; 2.7	ID02761.3p-miR	120	-138	93	24
FGFR2; 9.3	ID03047.3p-miR	48	-132	89	24
HIF1A; 16.0	miR-6789-5p	54	-132	90	24
JUN; 47.0	ID02008.5p-miR	269	-123	91	22
KRAS; 23.6	ID01310.3p-miR	17, 29	-121, -123	92,94	22
	ID03332.3p-miR	37	-132	89	24
LGALS3; 36.9	ID00329.3p-miR	21	-125	91	22
MMP2; 20.5	ID00278.3p-miR	110	-123	89	23
	ID03345.5p-miR	124	-127	90	24
NFKB1; 14.6	ID02064.5p-miR	128	-132	91	23
POU5F1; 6.3	miR-1273a	181	-119	90	25

	miR-1273g-3p	203	-113	96	21
	miR-1273d	237	-119	87	25
PTEN; 17.8	ID01310.3p-miR	531	-119	90	22
	ID02761.3p-miR	533	-132	89	24
	ID03037.3p-miR	536	-121	90	22
SFRP1; 11.0	ID01641.3p-miR	111	-132	89	24
SMAD2; 26.3	ID01492.3p-miR	16	-129	100	23
TGFB1; 12.8	ID00457.3p-miR	209	-129	95	22
	ID02064.5p-miR	211	-132	91	23
THBS1; 46.5	miR-328-5p	84	-123	89	23
	miR-6786-5p	88	-119	93	21
	ID01652.3p-miR	113	-129	92	23
TIMP2; 24.2	ID01520.3p-miR	60	-123	92	21
	ID00252.5p-miR	61	-134	90	24
	ID02668.5p-miR	219	-127	88	24
	ID00961.3p-miR	239	-132	93	23
	ID00049.5p-miR	269	-136	90	24
	ID00417.3p-miR	278	-123	94	21
	ID01293.5p-miR	281	-127	94	22
TIMP3; 22.9	ID02903.3p-miR	1102	-121	90	22
TYMS; 4.2	ID00252.5p-miR	87	-132	89	24

Table S3: The characteristics of the miRNA interactions with CDS mRNAs of gastric cancer candidate genes

Gene; NX	miRNA	Start of site, nt	ΔG, kJ/mole	ΔG/ΔGm, %	Length, nt
ACE; 1.9	ID00522.5p-miR	62	-125	89	23
	ID02294.5p-miR	64	-132	90	24
ARID1A; 19.5	ID01473.3p-miR	1093	-125	89	23
	ID03167.3p-miR	1399	-123	91	23
	ID01508.5p-miR	1459	-129	90	23
CDX2; 0.1	ID01895.5p-miR	416	-138	93	24
	ID00522.5p-miR	419	-125	89	23
	ID02052.5p-miR	608	-136	91	24
	ID01041.5p-miR	609	-129	88	24
	miR-3960	613	-117	93	20
EPHB2; 2.3	miR-4253	1089	-102	100	18
IGFBP3; 8.3	ID02982.3p-miR	435	-123	100	21
MUC1; 105.0	ID00645.5p-miR	507	-110	93	20
	miR-6752-5p	510	-119	90	22
MUC6; 76.6	miR-6815-3p	1218	-104	91	21
	ID02841.5p-miR	1238	-108	93	20
	miR-939-5p	1330	-121	88	24
OGG1; 10.8	ID00920.5p-miR	882	-134	94	24
PARP1; 15.9	ID01616.3p-miR	1275	-119	90	23
PGC; 963.7	ID01303.5p-miR	715	-115	92	20
SIRT1; 13.2	miR-4767	236	-134	94	23
	ID01560.3p-miR	264	-123	89	23
	ID03332.3p-miR	280, 287	-132, 138	89, 93	24
	ID00278.3p-miR	294	-123	89	23
	ID00811.3p-miR	300	-125	88	24

Table S4: The characteristics of the miRNA interactions with 3'UTR mRNAs of gastric cancer candidate genes

Gene; NX	miRNA	Start of site, nt	ΔG, kJ/mole	ΔG/ΔGm, %	Length, nt
AQP3; 3.9	miR-466	1255	-106	91	23
	ID00436.3p-miR	1255	-104	89	23
	ID01030.3p-miR	1259, 1269	-108	89	23
BRCA1; 5.1	miR-5095	6406	-106	91	21
	miR-619-5p	6412	-119	98	22
	miR-5096	6486	-110	98	21
	miR-5585-3p	6554	-110	95	22
CASP10; 8.7	miR-1273h-5p	2234	-106	91	21
	miR-1285-3p	2357	-106	91	22
	miR-1273g-3p	2589	-108	93	21
	miR-1273d	2623	-119	87	25
	miR-1273h-5p	2623	-117	100	21
	miR-619-5p	3247	-113	93	22
	miR-5585-3p	3389	-108	93	22
CCND1; 19.2	miR-574-5p	2593÷2597 (3)	-108÷-113	89÷93	23
	ID00470.5p-miR	2595, 2597	-108	89	23
CDH1; 21.6	miR-1273c	3251	-110	91	22
	miR-1273g-3p	3271	-108	93	21
	miR-1273h-5p	3305	-113	96	21
CDH17; 0.3	miR-1273a	2773	-119	90	25
	miR-1273g-3p	2795	-110	95	21
	miR-1273d	2829	-121	89	25
CDKN1A; 25.2	miR-4433-3p	1601	-106	91	21
CEACAM5; 20.1	miR-5585-3p	2441	-108	93	22
	miR-5095	3229	-115	98	21
	miR-619-5p	3235	-119	98	22
	miR-5585-3p	3378	-113	96	22
CLDN18; 126.9	miR-5095	1852	-106	91	21
	miR-619-5p	1858	-115	95	22
	miR-1273h-3p	2053	-113	90	22
CREB1; 13.3	miR-5095	2791	-110	95	21
	miR-619-5p	2797	-119	98	22
	miR-5096	2871	-106	94	21
	miR-619-5p	2932	-110	91	22
	miR-5585-3p	2939	-108	93	22
ERBB3; 32.9	miR-619-5p	4950	-117	96	22
	miR-619-5p	5104	-121	100	22
HPSE; 2.4	miR-1273g-3p	2081	-106	91	21
	miR-1273g-3p	2376	-108	93	21
	miR-5095	2759	-106	91	21
	miR-619-5p	2765	-110	91	22
	miR-5096	2839	-110	98	21
IL10; 2.0	miR-5095	1210	-115	98	21
	miR-619-5p	1216	-119	98	22
	miR-5096	1290	-106	94	21
IRF1; 17.5	miR-5095	2229	-110	95	21
	miR-619-5p	2235	-115	95	22
	miR-619-5p	2523	-110	91	22
	miR-5096	2597	-110	98	21
	miR-5095	2653	-110	95	21

	miR-619-5p	2659	-119	98	22
	miR-5585-3p	2800	-110	95	22
KRAS; 23.6	ID03224.5p-miR	3163	-121	92	23
	miR-1273g-3p	3176	-108	93	21
	miR-1273f	3209	-104	100	19
LEP; 0.2	miR-5095	3094	-110	95	21
	miR-619-5p	3100	-119	98	22
	miR-5096	3172	-104	92	21
	miR-5585-3p	3240	-108	93	22
MACC1; 5.0	ID01030.3p-miR	2962, 2976	-108	89	23
	ID01727.5p-miR	2963÷2981 (3)	-104	89	23
	miR-466	2966÷2990 (4)	-104÷-106	89÷91	23
	ID01030.3p-miR	3949÷3965 (8)	-108÷-110	89÷91	23
	miR-466	3951÷3967 (9)	-106÷-108	91÷93	23
	ID00436.3p-miR	3953÷3967 (8)	-104÷-106	89÷91	23
	miR-619-5p	4856	-117	96	22
	miR-1273f	5717	-102	98	19
	miR-1273d	5718	-123	91	25
MDM2; 9.8	miR-1273g-3p	2117	-113	96	21
	miR-1273g-3p	2486	-106	91	21
	miR-1273e	2521	-108	93	22
	miR-1273g-3p	6739	-113	96	21
MTHFR; 13.6	miR-5095	6855	-110	95	21
	miR-619-5p	6861	-115	95	22
	miR-5585-3p	7003	-110	95	22
MUC6; 76.6	ID01401.3p-miR	7895	-108	91	21
PDCD4; 27.6	miR-5095	3215	-106	91	21
	miR-619-5p	3221	-121	100	22
	miR-619-5p	3355	-110	91	22
PRKAA1; 22.1	miR-1273g-3p	2260	-110	95	21
	miR-1273f	2293	-102	98	19
	miR-1273d	2294	-121	89	25
	ID01404.5p-miR	2297	-113	91	23
	miR-1273e	2303	-106	91	22
SMAD4; 19.3	ID01838.5p-miR	4291	-113	90	24
	miR-1273g-3p	4312	-110	95	21
	ID01656.3p-miR	4342	-115	89	23
	miR-1273d	4346	-119	87	25
	ID01404.5p-miR	4349	-115	93	23
	ID02732.3p-miR	7721	-123	91	23
	miR-574-5p	7740÷7756 (8)	-108÷-113	89÷93	23
	ID00470.5p-miR	7744÷7752 (5)	-108	89	23
	ID00106.5p-miR	7825	-106	91	22
	miR-574-5p	7830	-108	89	23
STAT3; 29.4	miR-5095	3125	-106	91	21
	miR-619-5p	3131	-119	98	22
	miR-5585-3p	3268	-110	95	22
TGFB1; 12.8	miR-6089	2060, 2065	-132, -136	89, 91	24
	ID03306.3p-miR	2060	-123	94	21
	ID01382.3p-miR	2062	-113	93	20
	ID03208.5p-miR	2066	-125	88	24
	miR-4651	2087	-113	95	20
	ID00978.5p-miR	2089	-119	90	22
	ID00296.3p-miR	2093	-140	89	25

	miR-1183	4361	-132	90	27
TIMP2; 24.2	ID01941.5p-miR	1427	-117	89	24
TIMP3; 22.9	miR-1224-5p	3268	-104	96	19
TP53; 12.0	ID00548.3p-miR	1393	-115	89	23
	ID02379.3p-miR	1397	-119	89	24
	miR-1273c	2297	-110	91	22
	miR-1285-3p	2301	-110	95	22
	miR-1273g-3p	2317	-106	91	21
	miR-1273h-5p	2351	-106	91	21
	ID01838.5p-miR	2460	-110	88	24
	ID00785.5p-miR	2520	-113	90	23

```
GGGAGCAGCUGAGCCGCCGGCGCCUCGGCCGCCGCCGCCUCCUCCUCCGCCGCCGCCAGCCCGGAGCCUGAGCCGG hsa
GGGAGCAGCUGAGCCGCCGGCGCCUCGGCCGCCGCCGCC---UCCUCCUCCGCCGCCGCCAGCCCGGAGCCUGAGCCGG ggo
GGGAGCAGCUGAGCCGCCGGCGCCUCGGCCGCCGCCGCC---UCCUCCUCCGCCGCCGCCAGCCCGGAGCCUGAGCCGG ptr
GGGAGCAGCUGAGCCGCCGGCGCCUCGGCCGCCGCCGCC---UCCUCCUCCGCCGCCGCCAGCCCGGAGCCUGAGCCGG ppa
GGGAGCAGCUGAGCCGCCGGCGCCUCGGCCGCCGCCGCC---UCCUCCUCCGCCGCCGCCAGCCCGGAGCCUGAGCCGG pan
GGGAGCAGCUGAGCCGCCGGCGCCUCGGCCGCCGCCGCC---UCCUCCUCCGCCGCCGCCAGCCCGGAGCCUGAGCCGG nle
GGGAGCAGCUGAGCCGCCGGCGCCUCGGCCGCCGCCGCC---UCCUCCUCCGCCGCCGCCAGCCCGGAGCCUGAGCCGG mml
GGGAGCAGCUGAGCCGCCGGCGCCUCGGCCGCCGCCGCC---UCCUCCUCCGCCGCCGCCAGCCCGGAGCCUGAGCCGG mne
```

Figure S1: The nucleotide sequences of mRNA regions of orthologous *ARID1A* genes containing clusters of BSs of ten miRNAs.

```
GGCCUGCCGCCAUGGGCCCGCGCCGCCGCCGCCGCCUGUCACCCGGGCCGCGCGG hsa
GGCCUGCCGCCAUGGGCCCGCGCCGCCGCCGCCGCCUGUCACCCGGGCCGCGCGG ggo
GGCCUGCCGCCAUGGGCCCGCGCCGCCGUCGCCGCCUGUCACCCGGGCCGCGCGG ptr
GGCCUGCCGCCAUGGGCCCGCGCCGCCGUCGCCGCCUGUCACCCGGGCCGCGCGG ppa
GGCCUGCCGCCAUGGGCCCGCGCCGCCGCCGCCGCUUGUCACCCGGGCCGCGCGG pab
GGCCUGCCGCCAUGGGCCCGCGCCGCCGCCGCCGCCUGUCACCUGGGCCGCGCGG pan
GGCCUGCCGCCAUGGGCCCGCGCCGCCGCCGCCGCCUGUCACCCGGGCCGCGCGG nle
GGCCUGCCGCCAUGGGCCCGCGCCGCCGCCGCCGCCUGUCACCUGGGCCGCGCGG mml
GGCCUGCCGCCAUGGGCCCGCGCCGCCGCCGCCGCCUGUCACCUGGGCCGCGCGG mne
```

Figure S2: The nucleotide sequences of mRNA regions of orthologous *E2F1* genes containing clusters of BSs of three miRNAs.

```
CGCGGGCCACCCCCUGAAGUCGCCUGCCGCCGCCGCCGCACCUAGCGGACGGG hsa
CGCGGGCCACCCCCUGAAGUCGCCUGCCGCCGCCGC---ACCUAGCGGACGGG ggo
CGCGGGCCACCCCCUGAAGUCGCCUGCCGCCGCCGC---ACCUAGCGGACGGG ptr
CGCGGGCCACCCCCUGAAGUCGCCUGCCGCCGCCGC---ACCUAGCGGACGGG ppa
CCCGGGCCGCCCCCUGAAGUCGCCUGCCGCCGCCGC---ACCUAGCGGACGGG pab
CGCGGGCCGCCCCCUGAAGUCGCCUGCCGCCGCCGC---ACCUAGCGGACGGG nle
CGCAGGCCGCCCCCUGAAGUCGCCUGCCGCCGCCGC---ACCUAGCGGACGGG pan
CGCAGGCCGCCCCCUGAAGUCGCCUGCCGCCGCCGC---ACCUAGCGGACGGG mml
CGCAGGCCGCCCCCUGAAGUCGCCUGCCGCCGCCGC---ACCUAGCGGACGGG mne
CGCAGGCCGCCCCCUGAAGUCGCCUGCCGCCGCCGC---ACCUAGCGGACGGG mfa
```

Figure S3: The nucleotide sequences of mRNA regions of orthologous *TBC1D9* genes containing clusters of seven miRNA binding sites.

```
GCCGCCAGCAGCCUGGGCAACCCGCCGCCGCCGCCGCCCUCGGAGCUGAAGAAAGC hsa
GCCGCCAGCAGCCUGGGCAACCCGCCGCCGCCGCCGCCCUCGGAGCUGAAGAAAGC ggo
GCCGCCAGCAGCCUGGGCAACCCGCCGCCGCCGCCGCCCUCGGAGCUGAAGAAAGC pab
GCCGCCAGCAGCCUGGGCAACCCGCCGCCGCCGCCGCCCUCGGAGCUGAAGAAAGC ptr
GCCGCCAGCAGCCUGGGCAACCCGCCGCCGCCGCCGCCCUCGGAGCUGAAGAAAGC mml
GCCGCCAGCAGCCUGGGCAACCCGCCGCCGCCGCCGCCCUCGGAGCUGAAGAAAGC mne
GCCGCCAGCAGCCUGGGCAACCCGCCGCCGCCGCCGCCCUCGGAGCUGAAGAAAGC mfa
GCCGCCAGCAGCCUGGGCAACCCGCCGCCGCCGCCGCCCUCGGAGCUGAAGAAAGC nle
```

Figure S4: The nucleotide sequences of mRNA regions of orthologous *ARID1A* genes containing clusters of eight miRNA binding sites.

```
CUCCCACCGGCCCCGCGGCGCCCGCCGCCGGCCCCUGCGACCC hsa
CUCCCACCGGCCCCGCGGCGCCCGCCGCCGGCCCCUGCGACCC ggo
CUCCCACCGGCCCCGCGGCGCCCGCUGCCGGCCCCUGCGACCC ptr
CUCCCACCGGCCCCGCGGCGCCCGCUGCCGGCCCCUGCGACCC ppa
CUCCCACCGGCCCCGCGGCGCCCGCCGCCGGCCCCUGCGACCC pab
CUCCCACCGGCCCCGCGGCGCCAGCCGCCGGCCCCUGCGACCC pan
CUCCCACCGGCCCCGCGGCGCCAGCCGCCGGCCCCUGCGACCC mml
CUCCCACCGGCCCCGCGGCGCCAGCCGCCGGCCCCUGCGACCC mfa
CUCCCACCGGCCCCGCGGCGCCAGCCGCCGGCCCCUGCGACCC mne
CUCCCACCGGCCCCACGGCGCCCGCCGCCGGCCCCUGCGACCC nle
```

Figure S5: The nucleotide sequences of CDS mRNA regions of orthologous *E2F1* genes containing clusters of BSs of three miRNAs.

```
CCAACAUGGCGGCGGCGGCGGCGGCGGCGGCGGCGGCGGGCGCGGGCCCGGAGA ggo
CCAACAUGGCGGCGGCGGCGGCGGCGGCGGCGGCGG---GCGCGGGCCCGGAGA pab
CCAACAUGGCGGCGGCGGCGGCGGCGGGCGCGGGC------------CCGGAGA hsa
CCAACAUGGCGGCGGCGGCGGCGGCGGGCGCGGGC------------CCGGAGA ptr
CCAACAUGGCGGCGGCGGCGGCGGCGGGCGCGGGC------------CCGGAGA ppa
CCAACAUGGCGGCGGCGGCGGCGGCGGGCGCGGGC------------CCGGAGA pan
CCAACAUGGCGGCGGCGGCGGCGGCGGGCGCGGGC------------CCGGAGA mml
CCAACAUGGCGGCGGCGGCGGCGGCGGGCGCGGGC------------CCGGAGA mne
CCAACAUGGCGGCGGCGGCGGCGGCGGGCGCGGGC------------CCGGAGA mfa
CCAACAUGGCGGCGGCGGCGGCGGCGGGC------------------CCGGAGA mmu
CCAACAUGGCGGCGGCGGCGGCGGCGGGC------------------CCGGAGA rno
```

Figure S6: The nucleotide sequences of mRNA regions of orthologous *MAPK1* genes containing clusters of BSs of four miRNAs.

```
GGGAUGUCGCUGGGGGCCAAGGGCGCCGCCGGCCCUCUGCCCUCCGAGGCCGUGC hsa
GGGAUGUCGCUGGGGGCCAAGGGUGCCGCCGGCCCUCUGCCCUCCGAGGCCGUGC ggo
GGGAUGUCGCUGGGGGCCAAGGGUGCCGCCGGCCCUCUGCCCUCCGAGGCCAUGC ptr
GGGAUGUCGCUGGGGGCCAAGGGCGCCGCCGGCCCUCUGCCCUCCGAGGCCGUGC pab
GGGAUGUCGCUGGGGGCCAAGGGUGCCGCCGGCCCUCUGCCCUCCGAGGCCGUGC pan
GGGAUGUCGCUGGGGGCCAAGGGUGCCGCCGGCCCUCUGCCCUCCGAGGCCGUGC ppa
GGGAUGUCGCUGGGGGCCAAGGGUGCCGCCGGCCCUCUGCCCUCCGAGGCCGUGC nle
GGGAUGUCGCUGGGGGCCAAGGGCGCCGCCGGCCCUCUGCCCUCCGAGGCCGUGC mml
```

Figure S7: The nucleotide sequences of mRNA regions of orthologous *TERT* genes containing clusters of BSs of three miRNAs.

```
GGGUCCUCGCGAGGCGCCCGCCGCCGCCGCCGCCGCCGCCUUAGAGUCCGGA mne
GGGUCCUCGCGAGGCGCCCGCCGCCGCCGCCGCC---UUAGAGUCCGGA mml
GGGUCCUCGCGAGGCGCCCGCCGCCGCCGCC------UUAGAGUCCGGA pan
GGGUCCUCGCGAGGCGCCCGCCGCCGCCGCC------UUCGAGUCCGGA hsa
GGGUCCUCGCGAGGCGCCCGCCGCCGCC---------UUCGAGUCCGGA ggo
GGGUCCUCGCGAGGCGCCCGCCGCCGCC---------UUCGAGUCCGGA pab
GGGUCCUCGCGAGGCUCCCGCCGCCGCC---------UUCGAGUCCGGA nle
GGGUCCUCGCGAGGCGCCCGCCGCCGCC---------UUCGAGUCCGGA ptr
```

Figure S8: The nucleotide sequences of CDS mRNA regions of orthologous *VEGFC* genes containing clusters of seven miRNA binding sites.

<table>
<tr><td>*ATM*, miR-1273a,11054,-119,90
5'-**G**AGACAGAGUCUUGCU**C**UGUC**A**CCC-3'
|||| ||||||||||||||||||||
3'-**U**UCUUUCUCAGAACGA**A**ACAG**C**GGG-5'</td><td>*ATM*,miR-1273c,11056,-104,86
5'-GACAG**A**GUCU**U**G**C**U**C**UGUC**A**CC-3'
||||||||||||||||||||||
3'-CUGUC**C**CAGA**G**C**A**A**A**ACAG**C**GG-5'</td></tr>
<tr><td>*ATM*, miR-1273e,11119,-108,93
5'-UCU**G**C**C**UCCUGGGUUCAAGCAA-3'
|||||||||||||||||||||
3'-AGG**U**G**A**AGGACCCAAGUUCGUU-5'</td><td>*ATM*, miR-1273f,11109,-89,86
5'-CACUGAAACCUC**U**G**C**CUCC-3'
||||| ||||||||||||
3'-GUGACGUUGGAG**GUA**GAGG-5'</td></tr>
<tr><td>*ATM*,miR-1273g-3p,11076,-113,96
5'-C**C**CAGGCUGGAGUGCAGUGG**C**-3'
||||||||||||||||||||
3'-G**A**GUCCGACCUCACGUCACC**A**-5'</td><td>*CASP10*, miR-1273d,2623,-119,87
5'-ACUGCAGCCUU**G**ACCUCCC**A**GGUUC-3'
||||||||||||||||| |||||||
3'-UGACGUCGGAG**U**UGGAGUA**C**CCAAG-5'</td></tr>
<tr><td>*CASP10*,miR-1273f,2622,-89,86
5'-CACUGCA**G**CCUUGA**C**CUCC-3'
|||||||||||| ||||||
3'-GUGACGU**U**GGAGGU**A**GAGG-5'</td><td>*CASP10*,miR-1273g-3p,2589,-108,93
5'-C**CUG**GGCUGGAGUGCAGUGGU-3'
|||||||||||||||||||||
3'-G**AGU**CCGACCUCACGUCACCA-5'</td></tr>
<tr><td>*CASP10*,miR-1273h-5p,2234,-106,91
5'-ACUG**U**AG**U**CU**C**GACCUCCCAG-3'
||||||||||||||||||||
3'-UGAC**G**UC**G**GA**A**CUGGAGGGUC-5'</td><td>*CASP10*,miR-1273h-5p,2623,-117,100
5'-ACUGCAGCCUUGACCUCCCAG-3'
||||||||||||||||||||
3'-UGACGUCGGAACUGGAGGGUC-5'</td></tr>
<tr><td>*CDH1*,miR-1273g-3p,3271,-108,93
5'-G**C**CAGGCUGGAGUGCAGUGGU-3'
|||||||||||||||||||
3'-G**A**GUCCGACCUCACGUCACCA-5'</td><td>*CDH1*,miR-1273h-5p,3305,-113,96
5'-ACUGCAGCCUUGUCCUCCCAG-3'
||||||||||||| ||||||||
3'-UGACGUCGGAACUGGAGGGUC-5'</td></tr>
<tr><td>*CDH1*,miR-1273c,3251, -110,91
5'-GACAGGGUCUC**A**UU**C**U**A**UCGGCC-3'
|||||||||||||||||||| ||
3'-CUGUCCCAGAG**C**AA**A**A**C**AGC-GG-5'</td><td>*CDH17*,miR-1273a,2773, -119,90
5'-**G**AG**GUG**GAGUCUUGCU**C**UGUCGCCC-3'
|||| ||||||||||||||||||||
3'-**U**UC**UUU**CUCAGAACGA**A**ACAGCGGG-5'</td></tr>
<tr><td>*CDH17*,miR-1273d,2829,-121,89
5'-ACUGCA**A**CCUCC**G**CCUCCUGGGUUC-3'
||||||||||| ||||| |||||||
3'-UGACGU**C**GGAGU**U**GGAGUACCCAAG-5'</td><td>*CDH17*,miR-1273f,2828,-100,96
5'-CACUGCAACCUCC**GC**CUCC-3'
||||||||||||||||||
3'-GUGACGUUGGAGG**UA**GAGG-5'</td></tr>
<tr><td>*CDH17*,miR-1273g-3p,2795,-110,95
5'-CCCAGGCUGGAGUACAGUGGU-3'
||||||||||||||||||||
3'-GAGUCCGACCUCACGUCACCA-5'</td><td>*HPSE*,miR-1273c,2356, -104,86
5'-GACAGGGUAUCAUUCUGUUGCC-3'
|||||||| |||||||||||||
3'-CUGUCCCAGAGCAAAACAGCGG-5'</td></tr>
<tr><td>*HPSE*,miR-1273g-3p,2081,-106,91
5'-C**C**CAGGCUGGAGUGCC**A**UGGU-3'
||||||||||||||| |||||
3'-G**A**GUCCGACCUCACGU**C**ACCA-5'</td><td>*HPSE*,miR-1273g-3p,2376,-108,93
5'-C**C**CAGGCUUGAGUGCAGUGGU-3'
|||||||| |||||||||||
3'-G**A**GUCCGACCUCACGUCACCA-5'</td></tr>
<tr><td>*KRAS*,miR-1273e,3219,-102,87
5'-UCCA**U**CUCC**CA**GGUUCAAGC**GA**-3'
|||||||||||||||||||||
3'-AGGU**G**AAGG**AC**CCAAGUUCG**UU**-5'</td><td>*KRAS*,miR-1273f,3209, -104,100
5'-CACUGCAACCUCCAUCUCC-3'
||||||||||||||||||
3'-GUGACGUUGGAGGUAGAGG-5'</td></tr>
<tr><td>*KRAS*,miR-1273g-3p,3176,-108,93
5'-C**C**CAGGCUGGA**A**UGCAGUGG**C**-3'
||||||||||||||||||||
3'-G**A**GUCCGACCU**C**ACGUCACC**A**-5'</td><td>*KRAS*,miR-1273h-5p,3210,-100,85
5'-ACUGCA**A**CCU**C**CA**U**CUCCCAG-3'
||||||||||| |||||||||
3'-UGACGU**C**GGA**A**CU**G**GAGGGUC-5'</td></tr>
<tr><td>*MACC1*,miR-1273d,5718,-123,91
5'-ACUGCA**A**CCUCCACCUCCUGGGUUC-3'
||||||||||| ||||| |||||||
3'-UGACGU**C**GGAGUUGGAGUACCCAAG-5'</td><td>*MACC1*,miR-1273e,5727, -102,87
5'-UCCAC**C**UCCUGGGUUCAA**AAG**A-3'
|||||||||||||||||| ||
3'-AGGUG**A**AGGACCCAAGUU**C**G**U**U-5'</td></tr>
<tr><td>*MACC1*,miR-1273f,5717, -10,98
5'-CACUGCAACCUCCA**C**CUCC-3'
||||||||||||||||||
3'-GUGACGUUGGAGGU**A**GAGG-5'</td><td>*MACC1*,miR-1273g-3p,5684,-102,87
5'-C**C**CAG**A**CUGGAGUGC**G**GUGUU-3'
|||||||||||||||||| |
3'-G**A**GUC**C**GACCUCACG**U**CACCA-5'</td></tr>
<tr><td>*MACC1*,miR-1273g-5p.5714, -104,87
5'-ACU**C**ACUGCA**A**CCUCCACCUCC-3'
||||||||||||||| ||| ||
3'-UGA**A**UGACGU**C**GGAGUUGGUGG-5'</td><td>*MDM2*,miR-1273a,2795, -113,85
5'-**G**AGACAGAGUCUUGCU**CCA**UC**A**CCC-3'
|||| ||||||||||||||||||||
3'-**U**UCUUUCUCAGAACGA**AAC**AG**C**GGG-5'</td></tr>
<tr><td>*MDM2*,miR-1273c, 3215, -104,86
5'-GACAGGGUCUCCCUGUGU**U**GCC-3'
||||||||||| || |||||||
3'-CUGUCCCAGAGCAAAACA**G**CGG-5'</td><td>*MDM2*,miR-1273e,2521,-108,93
5'-UCC**G**CUUCC**C**GGGUUCAAGCCA-3'
|||||||||||||||||||| |
3'-AGG**U**GAAGG**A**CCCAAGUUCGUU-5</td></tr>
<tr><td>*MDM2*,miR-1273e,2860,-104,89
5'-UCC**G**C**CUU**CUGGGUUCAAGCUA-3'
|||||||||||||||||||| |
3'-AGG**U**G**AAG**GACCCAAGUUCGUU-5'</td><td>*MDM2*,miR-1273f,2850,-91,88
5'-CACUGCAAC**U**UCC**GC**CU**U**C-3'
||||||||||||||||||
3'-GUGACGUUG**G**AGG**UA**GA**G**G-5'</td></tr>
<tr><td>*MDM2*,miR-1273f, 6772, -96,92
5'-CACUGCAACCUC**UGC**CUCC-3'
||||||||||||||||||
3'-GUGACGUUGGAG**GUA**GAGG-5'</td><td>*MDM2*,miR-1273g-3p,2117,-113,96
5'-C**C**CAGGCUGGAGUGCAGUGG**C**-3'
||||||||||||||||||||
3'-G**A**GUCCGACCUCACGUCACC**A**-5'</td></tr>
<tr><td>*MDM2*,miR-1273g-3p,2486, -106,91
5'-C**C**CAGGCUGGAGUGCAGUGU**C**-3'
|||||||||||||||||| |
3'-G**A**GUCCGACCUCACGUCACC**A**-5'</td><td>*MDM2*,miR-1273g-3p,6739, -113,96
5'-CUCAGGCUGGAGU**A**CAGUGGU-3'
||||||||||||||||||||
3'-GAGUCCGACCUCA**C**GUCACCA-5'</td></tr>
<tr><td>*PRKAA1*,miR-1273d, 2294,-121,89
5'-ACUGCA**A**CCUCCACCUCC**C**GGGUUC-3'
||||||||||| ||||| |||||||
3'-UGACGU**C**GGAGUUGGAGU**A**CCCAAG-5'</td><td>*PRKAA1*, miR-1273e,2303,-106,91
5'-UCCAC**C**UCC**C**GGGUUCAAG**U**GA-3'
|||||||||||||||||||||
3'-AGGUG**A**AGG**A**CCCAAGUUC**G**UU-5'</td></tr>
<tr><td>*PRKAA1*,miR-1273f,2293,-102,98
5'-CACUGCAACCUCCA**C**CUCC-3'
||||||||||||||||||
3'-GUGACGUUGGAGGU**A**GAGG-5'</td><td>*PRKAA1*,miR-1273g-3p,2260,-110,95
5'-C**C**CAGGCUGGAGUGCA**A**UGGU-3'
||||||||||||||||||||
3'-G**A**GUCCGACCUCACGU**C**ACCA-5'</td></tr>
<tr><td>*PRKAA1*,miR-1273g-5p,2290.-102,86
5'-**G**CU**C**ACUGCA**A**CCUCCACCUCC-3'
||||||||||||||| ||| ||
3'-**U**GA**A**UGACGU**C**GGAGUUGGUGG-5'</td><td>*PRKAA1*,miR-1273h-5p,2294,-102,87
5'-ACUGCA**A**CCU**C**CACCUCCC**G**G-3'
||||||||||| |||||||||
3'-UGACGU**C**GGA**A**CUGGAGGG**U**C-5'</td></tr>
<tr><td>*SGCB*,miR-1273a,3312,-113,85
5'-**G**AGACAGAGUCUUG**UUC**UGUCGCCA-3'
|||| ||||||||||||||||||||
3'-**U**UCUUUCUCAGAAC**GAA**ACAGCGGG-5'</td><td>*SGCB*,miR-1273c,3314, -110,91
5'-GACAG**A**GUCU**U**GUU**C**UGUCGCC-3'
||||||||||||||||||||||
3'-CUGUC**C**CAGA**G**CAA**A**ACAGCGG-5'</td></tr>
<tr><td>*SGCB*,miR-1273e,3383, -100,85
5'-UC**UG**C**C**UCC**CA**GGUUCAAGC**GA**-3'
|||||||||||||||||||||
3'-AG**GU**G**A**AGG**AC**CCAAGUUCG**UU**-5'</td><td>*SGCB*,miR-1273f 3373, -96,92
5'-CACUGCAACCUC**U**G**C**CUCC-3'
||||||||||||||||||
3'-GUGACGUUGGAG**GUA**GAGG-5'</td></tr>
<tr><td>*SGCB*,miR-1273g-3p,3333, -106,91
5'-G**C**CAGGCUGGAGUGCAGUGG**C**-3'
|||||||||||||||||||
3'-G**A**GUCCGACCUCACGUCACC**A**-5'</td><td>*SGCB*,miR-1273h-5p,3374, -102,87
5'-ACUGCA**A**CCU**C**UGCCUCCCAG-3'
||||||||||| |||||||||
3'-UGACGU**C**GGA**A**CUGGAGGGUC-5'</td></tr>
<tr><td>*TP53*,miR-1273a,2295, -113,85
5'-**G**AGACUG**G**GUCU**C**GCUUUGU**U**GCCC-3'
|||| |||||||||||||||||||
3'-**U**UCUUUC**U**CAGA**A**CGAAACA**G**CGGG-5'</td><td>*TP53*,miR-1273c,2297, -110,91
5'-GACUGGGUCUCG**C**UUUGU**U**GCC-3'
||| ||||||||||||||||||
3'-CUGUCCCAGAGC**A**AAACA**G**CGG-5'</td></tr>
<tr><td>*TP53*,miR-1273g-3p,2317,-106,91
5'-C**C**CAGGCUGGAGUGGAGUGG**C**-3'
|||||||||||||| ||||||
3'-G**A**GUCCGACCUCACGUCACC**A**-5'</td><td>*TP53*,miR-1273g-5p,2347, -102,86
5'-**G**CUUACUGCAGCCU**UUG**CCUCC-3'
||||||||||||||| ||| ||
3'-**U**GAAUGACGUCGGA**GUU**GGUGG-5'</td></tr>
<tr><td>*POU5F1*,miR-1273a,181*,-119,90
5'-**G**AGACAGAGUCUUGCU**C**UGUC**A**CCC-3'
|||| ||||||||||||||||||||
3'-**U**UCUUUCUCAGAACGA**A**ACAG**C**GGG-5'</td><td>*POU5F1*,miR-1273c,183*, -104,86
5'-GACAG**A**GUCU**U**G**C**U**C**UGUC**A**CC-3'
||||||||||||||||||||||
3'-CUGUC**C**CAGA**G**C**A**A**A**ACAG**C**GG-5'</td></tr>
<tr><td>*POU5F1*,miR-1273d,237*,-119,87
5'-ACUGCA**A**CCUCC**G**CCUCC**C**GGGUUC-3'
||||||||||| ||||| |||||||
3'-UGACGU**C**GGAGU**U**GGAGU**A**CCCAAG-5'</td><td>*POU5F1*,miR-1273e,246*, -104,89
5'-UCC**G**C**C**UCC**C**GGGUUCAAG**UG**A-3'
|||||||||||||||||||||
3'-AGG**U**G**A**AGG**A**CCCAAGUUC**GU**U-5'</td></tr>
<tr><td>*POU5F1*,miR-1273f,236*,-100,96
5'-CACUGCAACCUCC**GC**CUCC-3'
||||||||||||||||||
3'-GUGACGUUGGAGG**UA**GAGG-5'</td><td>*POU5F1*,miR-1273g-3p,203*,-113,96
5'-C**C**CAGGCUGGAGUGCAGUGG**C**-3'
||||||||||||||||||||
3'-G**A**GUCCGACCUCACGUCACC**A**-5'</td></tr>
<tr><td colspan="2">**Note.** The top line contains sequentially: gene; miRNA; the beginning of the binding site, nt; ΔG, kJ/mole; ΔG/ΔGm,%. * - the miRNA binding site is located in the 5'UTR. The top line of the diagram is the nucleotide sequence of the miRNA binding site; the bottom line is the nucleotide sequence of the miRNA. Non-canonical pairs are in bold.</td></tr>
</table>

Figure S9: The schemes of miR-1273 family interaction in the 3'UTR of mRNA of candidate gastric cancer genes

<table>
<tr><td>FLT1,miR-466,6907(12),-108,93
5'-AUGUGUGUGUGUGUGUGUGUGUGU-3'
|||||||| |||||||||||||||
3'-UACACACA-ACGCACAUACACAUA-5'</td><td>FLT1,ID00436.3p-miR,6907(12),-104,89
5'-GUGUGUGUGUGUGUGUGUGUGUG-3'
|||||||||||||||||||||||
3'-CACACACGCAUAUAUACACACAU-5'</td></tr>
<tr><td>FLT1,ID01030.3p-miR,6909(11),-110;91
5'-GUAUGUGUGUGUGUGUGUGUGUGU-3'
|||||||||||| |||||||||||
3'-CGUACACGUACA-ACACACACACA-5'</td><td>JAK2,miR-466,5184(9),-106,91
5'-GUGUGUGUGUGUGUGUGUGUGUGU-3'
|||||||| |||||||||||||||
3'-UACACACA-ACGCACAUACACAUA-5'</td></tr>
<tr><td>JAK2,ID00436.3p-miR,5184(9),-104,89
5'-GUGUGUGUGUGUGUGUGUGUGUG-3'
|||||||||||||||||||||||
3'-CACACACGCAUAUAUACACACAU-5'</td><td>JAK2,ID01030.3p-miR,5184(9),-108,89
5'-GUGUGUGUGUGUGUGUGUGUGUGU-3'
|||||||||||| |||||||||||
3'-CGUACACGUACA-ACACACACACA-5'</td></tr>
<tr><td>SP1,ID00436.3p-miR,4147(7),-104,89
5'-GUGUGUGUGUGUGUGUGUGUGUG-3'
|||||||||||||||||||||||
3'-CACACACGCAUAUAUACACACAU-5'</td><td>SP1,miR-466,4147(7),-106,91
5'-GUGUGUGUGUGUGUGUGUGUGUGU-3'
|||||||| |||||||||||||||
3'-UACACACA-ACGCACAUACACAUA-5'</td></tr>
<tr><td>SP1,ID01030.3p-miR,4147(7),-108,89
5'-GUGUGUGUGUGUGUGUGUGUGUGU-3'
|||||||||||| |||||||||||
3'-CGUACACGUACA-ACACACACACA-5'</td><td>MACC1,ID00436.3p-miR,2990(6),-106,91
5'-GUGUGUGUGUGUGUGUGUGUGUA-3'
|||||||||||||||||||||||
3'-CACACACGCAUAUAUACACACAU-5'</td></tr>
<tr><td>MACC1,ID01030.3p-miR,2976(6), -108,89
5'-GUGUGUGUGUGUCUGUGUGUGUGU-3'
|||||||||||| |||||||||||
3'-CGUACACGUACA-ACACACACACA-5'</td><td>MACC1,miR-466,2966(6),-104,89
5'-GUGUGUGUCUGUGUGUGUGUGUCU-3'
|||||||| ||||||||||||| |
3'-UACACACA-ACGCACAUACACAUA-5'</td></tr>
<tr><td>MACC1,ID00436.3p-miR,3951(10),-106,91
5'-GUGUGUGUGUGUGUGUGUGUGUA-3'
|||||||||||||||||||||||
3'-CACACACGCAUAUAUACACACAU-5'</td><td>MACC1,ID01030.3p-miR,3949(11),-110,91
5'-ACAUGUGUGUGUGUGUGUGUGUGU-3'
|||||||||||| |||||||||||
3'-CGUACACGUACA-ACACACACACA-5'</td></tr>
<tr><td>MACC1,miR-466,3949(10),-108,93
5'-AUGUGUGUGUGUGUGUGUGUGUGU-3'
|||||||| |||||||||||||||
3'-UACACACA-ACGCACAUACACAUA-5'</td><td>AQP3,miR-466,1255,-106,91
5'-GUGUGUGUGUGCAUGUGUGUGCAU-3'
|||||||| |||||||||||||||
3'-UACACACA-ACGCACAUACACAUA-5'</td></tr>
<tr><td>AQP3,ID00436.3p-miR,1255,-104,89
5'-GUGUGUGUGUGCAUGUGUGUGCA-3'
|||||||||||||||||||||||
3'-CACACACGCAUAUAUACACACAU-5'</td><td>AQP3,ID01030.3p-miR,1259,-108,89,23
5'-GUGUGUGCAUGUGUGUGCAUGUGU-3'
|||||||||||| |||||||||||
3'-CGUACACGUACA-ACACACACACA-5'</td></tr>
<tr><td colspan="2">Note. The top line contains sequentially: gene; miRNA; the beginning of the binding site, nt; ΔG, kJ/mole; ΔG/ΔGm,%. * - the miRNA binding site is located in the 5'UTR. The top line of the diagram is the nucleotide sequence of the miRNA binding site; the bottom line is the nucleotide sequence of the miRNA. Non-canonical pairs are in bold.</td></tr>
</table>

Figure S10: The schemes of miR-466, ID01030.3p-miR, ID00436.3p-miR interaction with 3'UTR mRNA of gastric cancer candidate genes

<table>
<tr><td>CCND1,miR-574-5p,2595,-113,93
5'-ACACACACACACACACACACACACA-3'
|||||| ||||||||||||| ||
3'-UGUGUGAGUGUGUGUGUGUGAGU-5'</td><td>CCND1,ID00470.5p-miR,2595,-108,89
5'-ACACACAC**A**CA**C**ACACACA**C**ACA-3'
|||||||||||||||||||||||
3'-UGUGUGUG**C**GU**A**UGUGUG**C**AUGU-5'</td></tr>
<tr><td>IGF1,miR-574-5p,4042,-113,93
5'-ACACACACACACACACACACACACA-3'
|||||| ||||||||||||| ||
3'-UGUGUGAGUGUGUGUGUGUGAGU-5'</td><td>IGF1,ID00470.5p-miR,4042,-108,89
5'-ACACACAC**A**CACACACAC**AC**ACA-3'
|||||||||||||||||||||||
3'-UGUGUGUG**C**GUAUGUGUG**CA**UGU-5'</td></tr>
<tr><td>IGF2,miR-574-5p;2299;-106;88
5'-ACACACGCACACACA**UG**CACACA-3'
|||||| ||||||||||||| ||
3'-UGUGUGAGUGUGUGU**GU**GUGAGU-5'</td><td>IGF2,ID00470.5p-miR;2297;-113;93
5'-ACACACACGCA**C**ACACA**U**G**C**ACA-3'
|||||||||||||||||||||||
3'-UGUGUGUGCGU**A**UGUGU**G**C**A**UGU-5'</td></tr>
<tr><td>IGF2,miR-574-5p;2343;-106;88
5'-ACACACGCACACACA**U**GCACACA-3'
|||||| ||||||||||||| ||
3'-UGUGUGAGUGUGUGU**G**UGUGAGU-5'</td><td>IGF2,ID00470.5p-miR;2341;-113;93
5'-ACACACACGCA**C**ACACA**U**G**C**ACA-3'
|||||||||||||||||||||||
3'-UGUGUGUGCGU**A**UGUGU**G**C**A**UGU-5'</td></tr>
<tr><td>IGF2,miR-574-5p;2522;-108;89
5'-ACACA**U**GCACACACAGCACACACA - 3'
|||||| |||||||| ||||| ||
3'-UGUGU**G**AGUGUGUGU-GUGUGAGU - 5'</td><td>IGF2,ID00470.5p-miR;2520;-108;89
5'-ACACACA**U**GCA**C**ACACAGCA**C**ACA-3'
||||||||||||||||| ||||||
3'-UGUGUGU**G**CGU**A**UGUGU-GCAUGU-5'</td></tr>
<tr><td>IGF2,miR-574-5p;2835;-104;86
5'-ACACACA**UG**C**G**CACACACACGCA-3'
|||||| ||||||||||||| ||
3'-UGUGUGA**GU**G**U**GUGUGUGUGAGU-5'</td><td>IGF2,ID00470.5p-miR;2837;-110;91
5'-ACACA**UG**CGCA**C**ACACACG**C**ACA-3'
|||||||||||||||||||||||
3'-UGUGU**GU**GCGU**A**UGUGUGC**A**UGU-5'</td></tr>
<tr><td>SMAD4,miR-574-5p,7744,-113,93
5'-ACACACACACACACACACACACACA-3'
|||||| ||||||||||||| ||
3'-UGUGUGAGUGUGUGUGUGUGAGU-5'</td><td>SMAD4,ID00470.5p-miR,7744,-108,89
5'-ACACACAC**A**CA**C**ACACACA**C**ACA- 3'
|||||||||||||||||||||||
3'-UGUGUGUG**C**GUAUGUGUG**C**AUGU-5'</td></tr>
<tr><td>XRCC1,miR-574-5p,2035,-113,93
5'-ACACACACACACACACACACACACA-3'
|||||| ||||||||||||| ||
3'-UGUGUGAGUGUGUGUGUGUGAGU-5'</td><td>XRCC1,ID00470.5p-miR,2035,-108,89
5'-ACACACAC**A**CA**C**ACACACA**C**ACA-3'
|||||||||||||||||||||||
3'-UGUGUGUG**C**GU**A**UGUGUGCAUGU-5'</td></tr>
<tr><td>ZEB1,miR-574-5p,3587,-113,93
5'-ACACACACACACACACACACACACA-3'
|||||| ||||||||||||| ||
3'-UGUGUGAGUGUGUGUGUGUGAGU-5'</td><td>ZEB1,ID00470.5p-miR,3587, -108,89
5'-ACACACAC**A**CA**C**ACACAC**AC**ACA-3'
|||||||||||||||||||||||
3'-UGUGUGUG**C**GU**A**UGUGUG**CA**UGU-5'</td></tr>
<tr><td colspan="2">Note. The top line contains sequentially: gene; miRNA; the beginning of the binding site, nt; ΔG, kJ/mole; ΔG/ΔGm,%. * - the miRNA binding site is located in the 5'UTR. The top line of the diagram is the nucleotide sequence of the miRNA binding site; the bottom line is the nucleotide sequence of the miRNA. Non-canonical pairs are in bold.</td></tr>
</table>

Figure S11: The schemes of interaction of miR-574-5p and ID00470.5p-miR in the 3'UTR of mRNA of candidate gastric cancer genes
